# Grazing intensity drives above–belowground productivity trade-offs and reveals belowground dominance in temperate herbaceous marsh wetlands

**DOI:** 10.3389/fpls.2026.1773820

**Published:** 2026-03-19

**Authors:** Mingyue Pan, Linguang Luan, Yingqi Han, Qiong Han, Weihong Zhu, Jingzhi Wang

**Affiliations:** College of Geography and Ocean Sciences, Yanbian University, Yanji, China

**Keywords:** above ground-belowground productivity, carbon allocation trade-off, grazing intensity, herbaceous marsh wetland plant diversity, structural equation modeling

## Abstract

**Introduction:**

Livestock grazing, as a major anthropogenic disturbance, significantly affects plant species diversity and net primary productivity (NPP) in wetland ecosystems and their relationships with soil properties. However, how grazing intensity interacts with environmental factors to regulate the trade-off between aboveground and belowground productivity remains unclear.

**Methods:**

Field experiments were conducted in natural herbaceous marshes in the lower Tumen River basin of northeastern China. Three grazing intensity treatments were established: non-grazed (NG), moderately grazed (MG), and heavily grazed (HG). Aboveground net primary productivity (ANPP), belowground net primary productivity (BNPP), plant diversity indices, and soil physicochemical properties were measured. Structural equation modeling (SEM) was used to evaluate the direct and indirect pathways through which grazing intensity influences productivity, plant diversity, and soil properties.

**Results:**

Grazing altered plant community structure, shifting dominance from Cyperaceae to forb species, and moderate grazing significantly increased plant species diversity. With increasing grazing intensity, ANPP increased while BNPP decreased, indicating a clear aboveground–belowground productivity trade-off. Non-grazed plots exhibited the highest total NPP, suggesting that reductions in BNPP under grazing constrain ecosystem carbon accumulation. Grazing also reduced soil organic carbon, nutrient availability, moisture, and electrical conductivity while increasing bulk density and redox potential. SEM analysis showed significant direct effects of grazing on ANPP and BNPP, whereas indirect effects mediated by species diversity or soil properties were weak.

**Discussion:**

Although BNPP declined with increasing grazing intensity, it exerted the strongest control over total NPP, highlighting the critical role of belowground processes in regulating ecosystem carbon cycling under grazing disturbance. Moderate grazing can promote aboveground growth and diversity, whereas heavy grazing damages root systems and threatens ecosystem stability. These findings provide new insights into grazing–environment interactions and offer a scientific basis for the conservation and restoration of herbaceous marsh wetlands.

## Introduction

Wetlands are among the most productive and functionally diverse ecosystems globally, playing irreplaceable roles in biodiversity conservation, hydrological regulation, and carbon sequestration (Junk et al., 2013; Xu et al., 2020). Herbaceous marshes, a widespread wetland type particularly in temperate and subtropical regions, serve critical ecological functions. Dominated by species of Poaceae and Cyperaceae, and other herbaceous plants, these wetlands enhance soil structure, improve water retention, and accumulate organic carbon through extensive rhizome systems, establishing them as vital carbon sinks (Chmura et al., 2003). Plant-microbe interactions in herbaceous marshes facilitate nitrogen and phosphorus cycling, while their structural complexity provides essential habitats for birds, amphibians, and invertebrates, underpinning regional biodiversity (Peralta et al., 2016). However, anthropogenic pressures—including land reclamation, hydrological regulation, and intensive grazing—have driven vegetation degradation, invasive species proliferation, and functional decline in these ecosystems (Davidson, 2014). Compounding these threats, climate change-induced temperature increases and altered precipitation patterns exacerbate ecological vulnerabilities, destabilizing hydrological processes, carbon-nitrogen cycling, and biodiversity maintenance (Erwin, 2009; Junk et al., 2013). Understanding the ecological processes governing herbaceous marshes and their resilience to disturbances is therefore imperative for global ecological security and sustainable wetland management (Zedler and Kercher, 2005).

Livestock grazing represents a prevalent anthropogenic disturbance that significantly impacts wetland vegetation-soil systems, particularly in temperate and semi-humid regions (Greenwood and McKenzie, 2001; Wang et al., 2020). This disturbance modulates plant community assembly by altering species composition, functional group dominance hierarchies, and vegetation structural complexity, thereby redirecting ecosystem energy fluxes and nutrient cycling pathways (Bardgett and Wardle, 2003). Under moderate grazing intensities, observed ecological responses frequently conform to the Intermediate Disturbance Hypothesis (IDH), wherein suppression of competitive dominants and enhanced niche partitioning collectively elevate species diversity (Connell, 1978). Conversely, intensive grazing precipitates community dominance by ruderal or disturbance-adapted species, ultimately driving diversity-function decoupling and degradation of ecosystem multifunctionality (Flombaum and Sala, 2008). A critical knowledge gap persists in the disproportionate research focus on aboveground net primary productivity (ANPP), while neglecting belowground net primary productivity (BNPP)—the dominant carbon flux component in wetland ecosystems (Wardle et al., 2004). Chronic soil anoxia in wetlands decelerates root decomposition while accelerating turnover rates, positioning BNPP as a pivotal carbon sequestration mechanism (Jackson et al., 1997). Grazing-mediated BNPP responses frequently exhibit inverse relationships with ANPP, reflecting strategic resource allocation trade-offs in plants (De Deyn et al., 2008). Grazing further modulates root growth and belowground ecological processes through soil compaction, nutrient supply dynamics, and redox potential alterations (Zhou et al., 2016). Concurrently, fecal-urine deposition, microbial activity disturbances, and hydrological processes involving infiltration and salinity gradients collectively regulate wetland productivity dynamics (Hamilton and Frank, 2001).

Although grazing mechanisms have been extensively studied in grassland systems, the coupling mechanisms linking plant diversity, soil nutrients, and productivity under grazing pressure remain systematically underexplored in herbaceous swamp wetlands. These wetlands exhibit dual characteristics of grasslands and wetlands, with their unique hydrological regimes and soil nutrient cycling processes driving distinct response patterns to grazing disturbances compared to typical grassland ecosystems (Marty, 2005). Therefore, an integrated investigation of grazing impacts on plant communities, soil properties, and ecosystem functions is imperative. This study focuses on a representative herbaceous swamp wetland in Jingxin Town, Hunchun City, Yanbian Korean Autonomous Prefecture, Northeast China. We established grazing intensity gradients to address three key questions:(1) Response patterns: How do plant diversity, soil parameters, and aboveground/belowground net primary productivity (ANPP/BNPP) respond to varying grazing intensities? (2) Productivity trade-offs: Do ANPP and BNPP exhibit trade-offs or synergies, and how do these relationships shift with increasing grazing pressure? (3) Mechanistic pathways: What are the dominant pathways through which grazing influences productivity, as quantified by structural equation modeling (SEM) integrating plant diversity and soil factors? This research advances understanding of swamp wetlands’ hierarchical responses to grazing, particularly their divergence from grassland adaptation mechanisms. The findings will clarify carbon sequestration dynamics in grazed wetlands and inform adaptive management strategies to balance ecological conservation with pastoral livelihoods. Furthermore, the mechanistic insights contribute to regional ecological security and China’s “dual carbon” goals (carbon neutrality and peak carbon emissions) through evidence-based wetland stewardship.

## Materials and methods

### Study area

**Figure 1:**
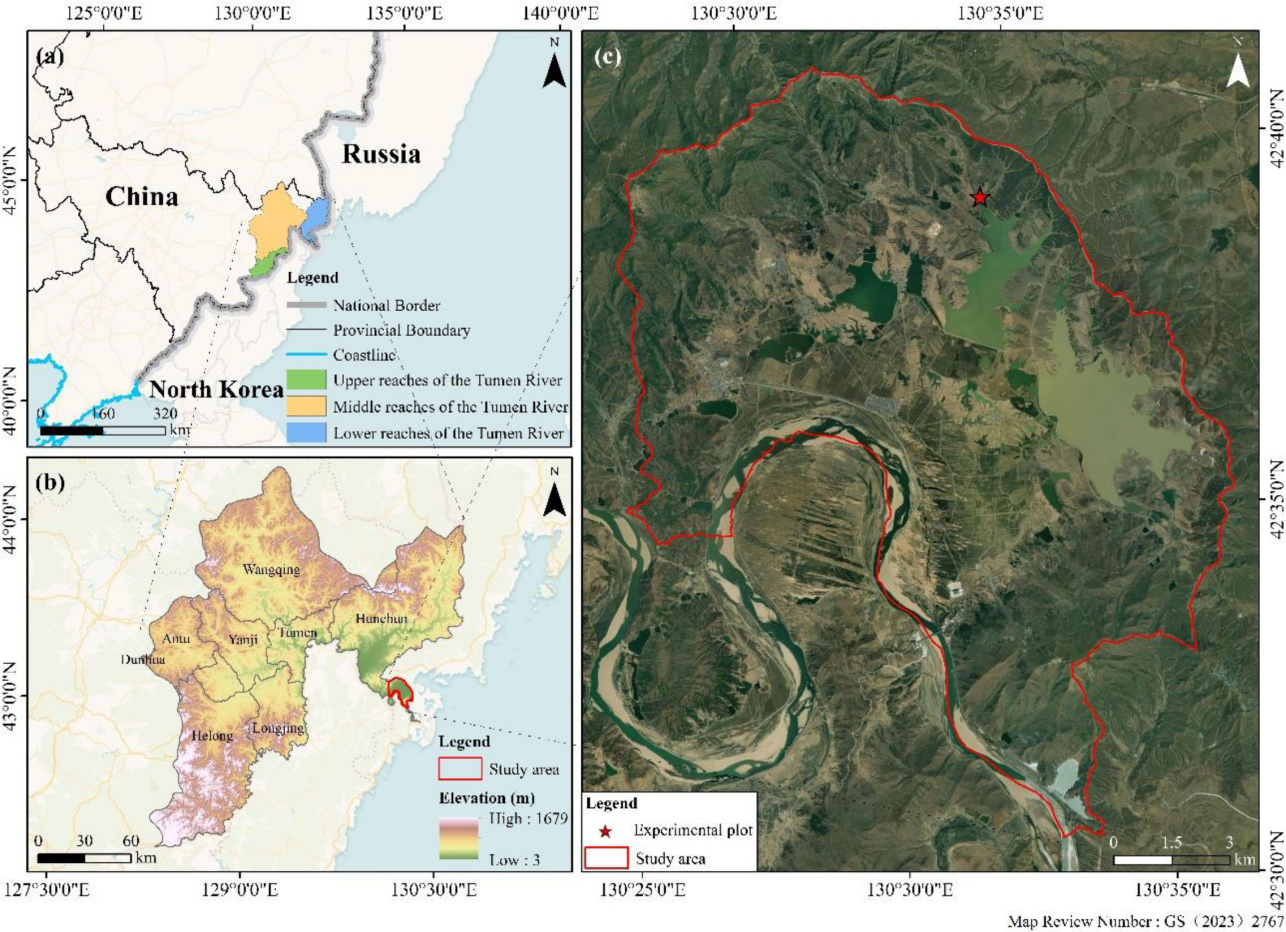
Map of sampling sites in the lower reaches of the Tumen river.

The Tumen River Basin is situated in southeastern Jilin Province, Northeast China (42°25′–43°30′N, 129°52′–131°18′E), bordering North Korea and Russia. As a core area of Northeast Asia, it serves as a strategic hub within the “Golden Triangle” economic zone of Northeast Asia (**Figure 1**). This basin supports diverse wetland ecosystems characterized by rich biodiversity, providing critical temporary habitats for endangered migratory waterbirds and rare flora or rare fauna. Consequently, it represents one of Northeast Asia’s most vital regions for biodiversity conservation (Zheng et al., 2017).

Jingxin Wetland, located in the southern part of Hunchun City, occupies the lower reaches of the Tumen River Basin at elevations of 5–15 m. Proximity to the Sea of Japan imposes a distinct temperate marginal maritime climate, exhibiting the following features relative to inland areas at equivalent latitudes: Milder winters and cooler summers; Intense seasonal monsoons with persistent cloud cover and low solar radiation; High annual precipitation (600–800 mm) and a mean annual temperature of 5.6 °C, classifying it as a humid zone (Wu et al., 2024).

### Experimental design and field sampling

#### Site selection and grazing gradient establishment

Field investigations were conducted from May to September 2024 in herbaceous marsh wetlands of Jingxin Town, lower Tumen River basin, Northeast China. Field investigations indicated that the herbaceous marsh wetlands in the study area have been subjected to livestock grazing since the early 21st century. The grazing experiment in this wetland was established in 2021 and has been ongoing since then. Grazing intensity was classified by integrating multiple indicators, including approximate livestock stocking density (assessed using camera traps to monitor livestock activity), the degree of aboveground biomass removal, and visible signs of soil disturbance caused by trampling. Based on these criteria, the study area was categorized into three grazing intensity levels: non-grazed (natural), moderately grazed, and heavily grazed sites (**Table 1**). For each treatment, five 10 m × 10 m plots were established, separated by at least 30 m. Prior to grazing, three movable exclosure cages (1 m × 1 m × 1 m) were randomly placed in each plot, totaling 45 cages.

**Table 1:** Description of grazing intensity levels in the study area.

Grazing intensity	Grazing intensity	Grazing intensity	Soil trampling
no grazing	no livestock activity	no removal	no trampling
moderate grazing	30–50 cattle freely grazing	about 50%	moderate trampling
heavy grazing	70–90 cattle freely grazing	about 70%	heavy trampling

#### Vegetation and soil sampling

In August, vegetation inside cages in NG plots was harvested (15 quadrats) to estimate aboveground net primary productivity (ANPP). In grazed plots, livestock consumption was monthly estimated as the difference in dry biomass between inside and outside cages, and ANPP was calculated as the sum of residual biomass and consumed biomass (Hao et al., 2022). Species diversity was assessed by recording species identity, cover, richness, and mean plant height (five individuals per species). Community cover (CC) and height (CH) were determined accordingly. All aboveground plant samples were oven-dried at 65 °C for 48 h and weighed for aboveground biomass (AGB).

Belowground biomass was sampled adjacent to each cage using a root auger (10 cm diameter, 0–30 cm depth). Roots were washed free of soil, separated into live and dead fractions (Pregitzer et al., 2002),oven-dried (65 °C, 48 h), and weighed. Belowground net primary productivity (BNPP) was calculated using a decision matrix method balancing live and dead root biomass (Wu et al., 2010). Soil sampling was conducted simultaneously, with five cores collected per plot (75 samples total). Samples were air-dried and ground. Soil organic carbon (SOC) and total nitrogen (TN) were determined using a Vario EL cube elemental analyzer, while ammonium nitrogen (NH_4_^+^-N), nitrate nitrogen (NO_3_^-^-N), and total phosphorus (TP) were measured with a Futura continuous flow analyzer. Soil pH was measured in a 1:2.5 soil-to-water suspension, and electrical conductivity (EC) and redox potential in a 1:5 suspension using a laboratory pH/EC meter.

#### Species diversity calculations

This experiment used three diversity indices in order to reflect changes in plant species diversity: (1) The Shannon-Wiener diversity index (H’), which quantifies overall species diversity by integrating both richness (number of species) and evenness (relative abundance distribution) through the logarithmic transformation of species proportion values (Shannon, 1948); (2) Pielou’s evenness index (J), calculated as the ratio of observed Shannon diversity to the theoretical maximum diversity for a given species count, serving as a standardized measure of abundance uniformity (Pielou, 1966); and (3) Margalef’s richness index (M), derived from species count and total individual abundance to evaluate taxonomic richness independent of sampling effort (Margalef, 1973). These indices collectively capture structural (e.g., dominant species) and functional (e.g., resource allocation) aspects of vegetation communities under grazing pressure. The three diversity indices are calculated as follows:


$$H'=-\sum_{i=1}^{s}P_{i}\text{ln}P_{i}$$

M=(S-1)/lnN

$$J=H^{\prime }/\text{ln}(\text{S})$$

where H′, M, and J represent the Shannon-Wiener, Margalef, and Pielou indices, respectively; Pi denotes the proportion of the total number of individual species of i species in the community; S indicates the total number of species; N indicates the total number of individuals.

The importance value (*IV*) for each species in a plant community was calculated as follows (Wang et al., 2022):


IV=(RC+RH+RD)/3

RC, RH, and RD are the relative coverage, relative height, and relative density.

#### Relative growth rate calculation

To assess vegetation growth dynamics under varying grazing intensities, the relative growth rate (RGR) of plant communities was calculated. RGR quantifies the proportional growth per unit of existing biomass over time, serving as a robust indicator of temporal growth vigor. The calculation follows:


$$\text{RGR}=({\text{lnW}}_{2}-{\text{lnW}}_{1})/({\text{t}}_{2}-{\text{t}}_{1})$$

where W1and W2​ represent the aboveground biomass (AGB) at times t_1_​ and t_2_, respectively, and (t_2_​−t_1_​) is the interval in months. This method minimizes the influence of initial biomass differences on growth rate comparisons, thereby providing a more accurate reflection of temporal variation in plant growth across grazing treatments (Ahmed et al., 2020).

### Data analysis

All analyses were conducted in R (version 4.3.2). Data were checked for normality (Shapiro–Wilk test) and homogeneity of variances (Levene’s test). One-way ANOVA was used to test the effects of grazing intensity on ANPP, BNPP, NPP, plant diversity indices, and soil physicochemical properties. Temporal changes in relative growth rate (RGR) were analyzed using repeated-measures ANOVA, with grazing intensity as a fixed factor and month as a repeated measure. When assumptions were violated or data were missing, linear mixed-effects models (LMMs) with plot as a random factor were applied to test grazing × time interactions. Significant effects were further examined using Tukey’s HSD *post hoc* tests (*P* < 0.05). Pearson correlations were performed to assess relationships among plant diversity, soil factors, and productivity indices. Structural equation modeling (SEM, lavaan package) was employed to explore potential pathways by which grazing affects productivity, including indirect effects mediated by plant diversity and soil nutrients. Model fit was evaluated using χ², RMSEA (< 0.08), CFI (> 0.90), TLI (> 0.90), and SRMR (< 0.08). Results are presented as mean ± SE, and all figures were generated with ggplot2.

## Results

### Effects of grazing intensity on plant community structure and diversity indices in herbaceous marsh wetlands

**Figure 2:**
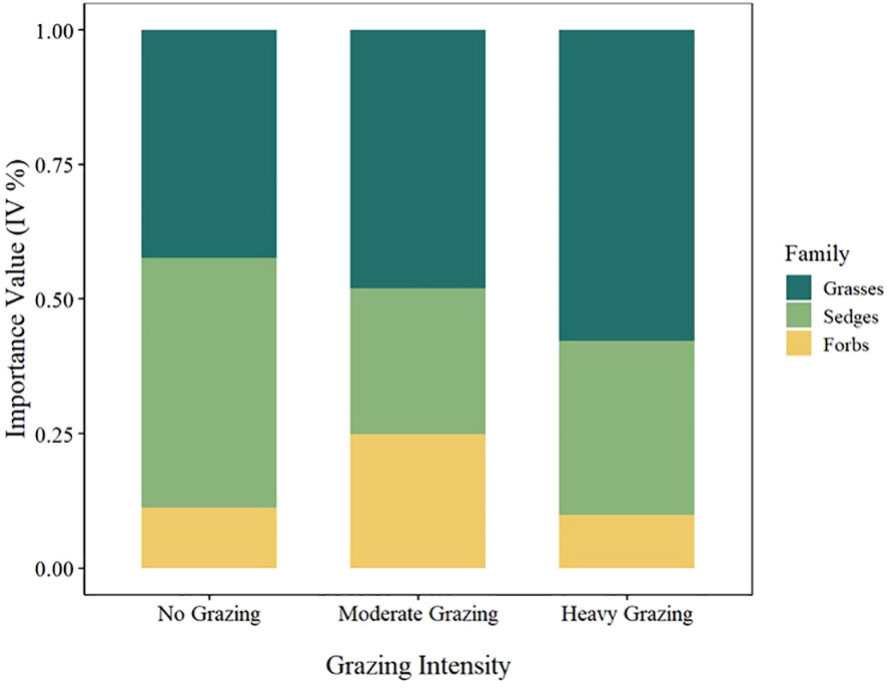
Distribution of importance values of plant functional groups under different grazing intensities; HG, heavy grazing; MG, moderate grazing; NG, no grazing.

Increased grazing intensity significantly altered the plant community composition and diversity indices in the herbaceous marsh wetlands. Our results revealed notable differences in the families and importance values of dominant species under varying grazing intensities (**Table 2**). The dominant species primarily belonged to the *Cyperaceae*, *Poaceae*, and several *forb* groups. Under heavy grazing (HG), species such as *Scirpus asiaticus Nakai* (*IV*=0.24) and *Eleocharis wichurae* (*IV*=0.05) from the *Cyperaceae* family exhibited high importance values. *Forbs* including *Thelypteris palustris* (*IV*=0.09), *Isodon inflexus* (*IV*=0.07), and *Polygonum hastato-sagittatum* (*IV*=0.06) also showed prominence under HG conditions. Under moderate grazing (MG), new dominant species emerged within the *Cyperaceae*, such as *Scirpus asiaticus* (*IV*=0.15) and *Heleocharis mamillata* (*IV*=0.06), while *Arthraxon hispidus* (*IV*=0.13) from the *Poaceae* family became dominant. *Forbs* such as *Potentilla fragarioides* (*IV*=0.04) and *Thelypteris palustris* (*IV*=0.03) demonstrated considerable adaptability. In the no-grazing (NG) treatment, *Cyperaceae* species including *Carex appendiculata* (*IV*=0.43) and *Carex heishuiensis* (*IV*=0.03) displayed high importance values, indicating their ecological dominance under low-disturbance conditions. *Forbs* such as *Calla palustris L.* (0.1251), *Polygonum sagittatum* (*IV*=0.03), and *Artemisia manshurica* (*IV*=0.02) also exhibited certain competitiveness. The *Poaceae* family was most competitive under MG, representing the dominant group under moderate disturbance. *Forbs* were prominent across all three grazing conditions, reflecting their broad ecological adaptability (**Figure 2**).

**Table 2:** Dominant species, families, and importance values in herbaceous marsh wetlands under different grazing intensities (NG, ungrazed; MG, moderate grazing; HG, heavy grazing).

Families	Species	NG	MG	HG
Cyperaceae	*Carex appendiculata*	0.43	*——*	——
	*Carex heishuiensis*	0.03	*——*
	*Scirpus asiaticus*	*——*	0.15	*——*
	*Heleocharis mamillata*	*——*	0.06	*——*
	*Eleocharis wichurae Boeckeler*	*——*	0.04	*——*
	Scirpus asiaticus Nakai	*——*	*——*	0.24
	*Eleocharis wichurae Boeckeler*	*——*	*——*	0.05
	*Carex rostrata Stokes in Withering*	*——*	*——*	0.03
Poaceae	*Panicum bisulcatum Thunb.*	0.05	0.07	0.04
	*Digitaria sanguinalis (L.) Scop*	0.04	*——*	*——*
	*Arthraxon hispidus*	*——*	0.13	*——*
Grasses	*Calla palustris L.*	0.12	*——*	*——*
	*Triadenum japonicum (Blume) Makino*	0.04	*——*	*——*
	*Iris sanguinea*	0.04	*——*	*——*
	*Polygonum sagittatum*	0.03	*——*	*——*
	*Galium pauciflorumBge.*	0.03	*——*	*——*
	*Polygonum maackianum Regel*	0.03	*——*	*——*
	*Potentilla fragarioides Linn.*	*——*	0.03	0.05
	*Lythrum salicaria Linn.*	*——*	0.03	*——*
	*Thelypteris palustris (Salisb.) Schott*	*——*	0.03	*——*
	*Rabdosia inflexa (Thunb.) Hara*	*——*	0.03	*——*
	*Pilea pumila (L.) A. Gray* var. *pumila*	*——*	0.03	*——*
	*Thelypteris palustris (Salisb.) Schott*	*——*	*——*	0.09
	*Isodon inflexus*	*——*	*——*	0.07
	*Polygonum hastato-sagittatum Makino*	*——*	*——*	0.06
	*Geum aleppicum Jacq.*	*——*	*——*	0.03
	*Artemisia manshurica Kom.*	*——*	*——*	0.02

**Figure 3:**
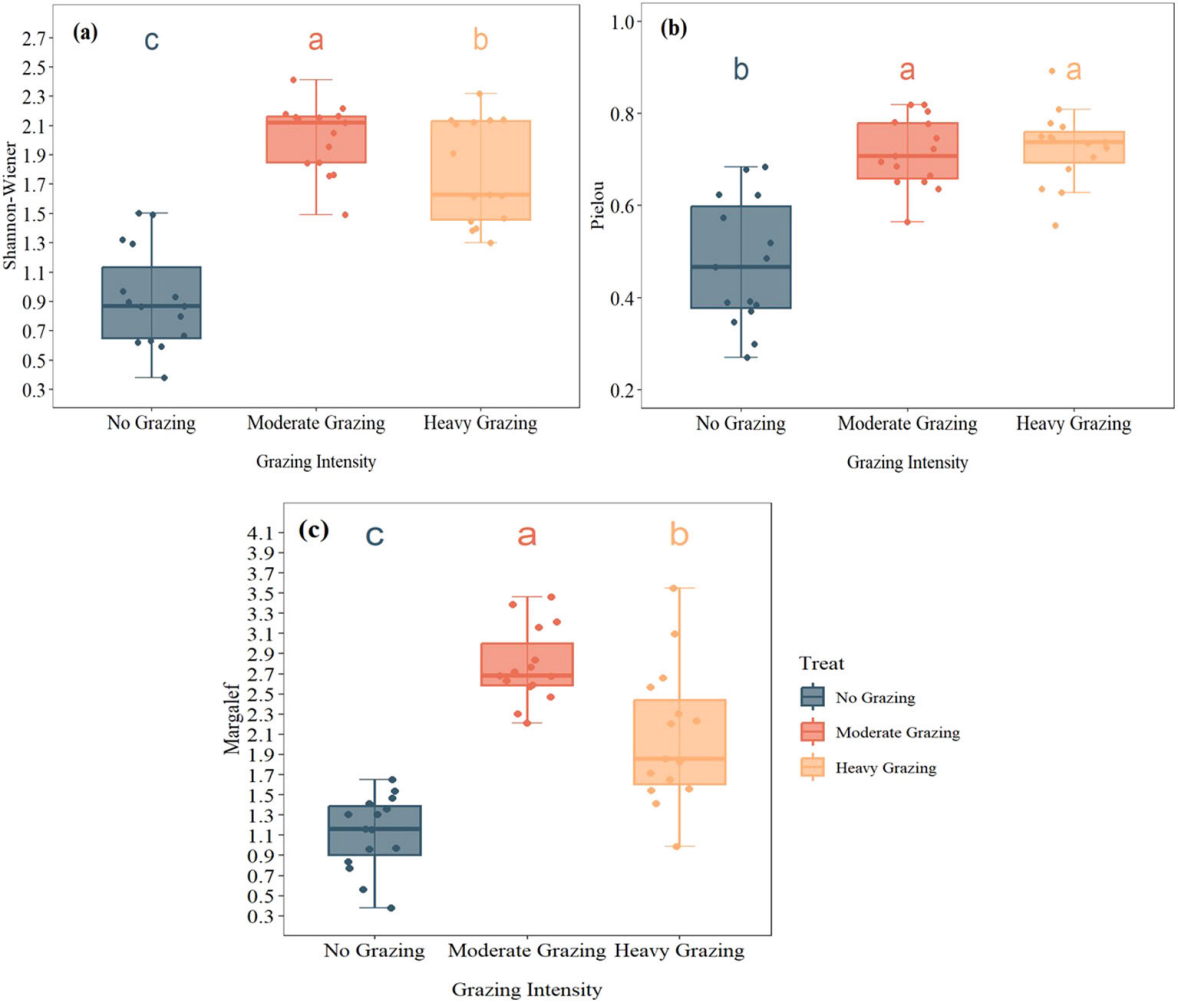
Effects of grazing intensity on community diversity indices: **(A)** Shannon–Wiener index, **(B)** Pielou evenness index, and **(C)** Margalef richness index under heavy grazing (HG), moderate grazing (MG), and no grazing (NG). Different lowercase letters above the boxes indicate significant differences among treatments at *P* < 0.05.

One-way ANOVA results indicated that grazing intensity had a significant effect on plant community diversity indices (**Figure 2**). Significant differences were observed in the Shannon-Wiener diversity index(Shannon-Wiener: F_2,42_ = 50.62, *P* < 0.001), Pielou evenness index(Pielou: F_2,42_ = 29.66, *P* < 0.001), and Margalef richness index(Margalef: F_2,42_ = 42.13, *P* < 0.001) across different grazing intensities. Compared to the NG treatment, both HG and MG treatments significantly increased the Shannon-Wiener index (F_2,42_ = 50.62, *P* < 0.001), with MG being significantly higher than HG (**Figure 3A**). The Pielou evenness index also differed significantly among grazing intensities (F_2,42_ = 29.66, *P* < 0.001). Although no significant difference was detected between MG and HG, both were significantly higher than NG (**Figure 3B**). The Margalef richness index showed a trend similar to the Shannon-Wiener index (F_2,42_ = 42.13, *P* < 0.001), with the highest species richness observed in MG, followed by HG, while NG had the lowest species richness (**Figure 3C**). Comprehensive analysis suggests that moderate grazing (MG) promotes community species diversity, whereas the no-grazing (NG) condition results in the lowest level of community diversity.

### Responses of net primary productivity and relative growth rate to different grazing intensities in a herbaceous marsh wetland

**Figure 4:**
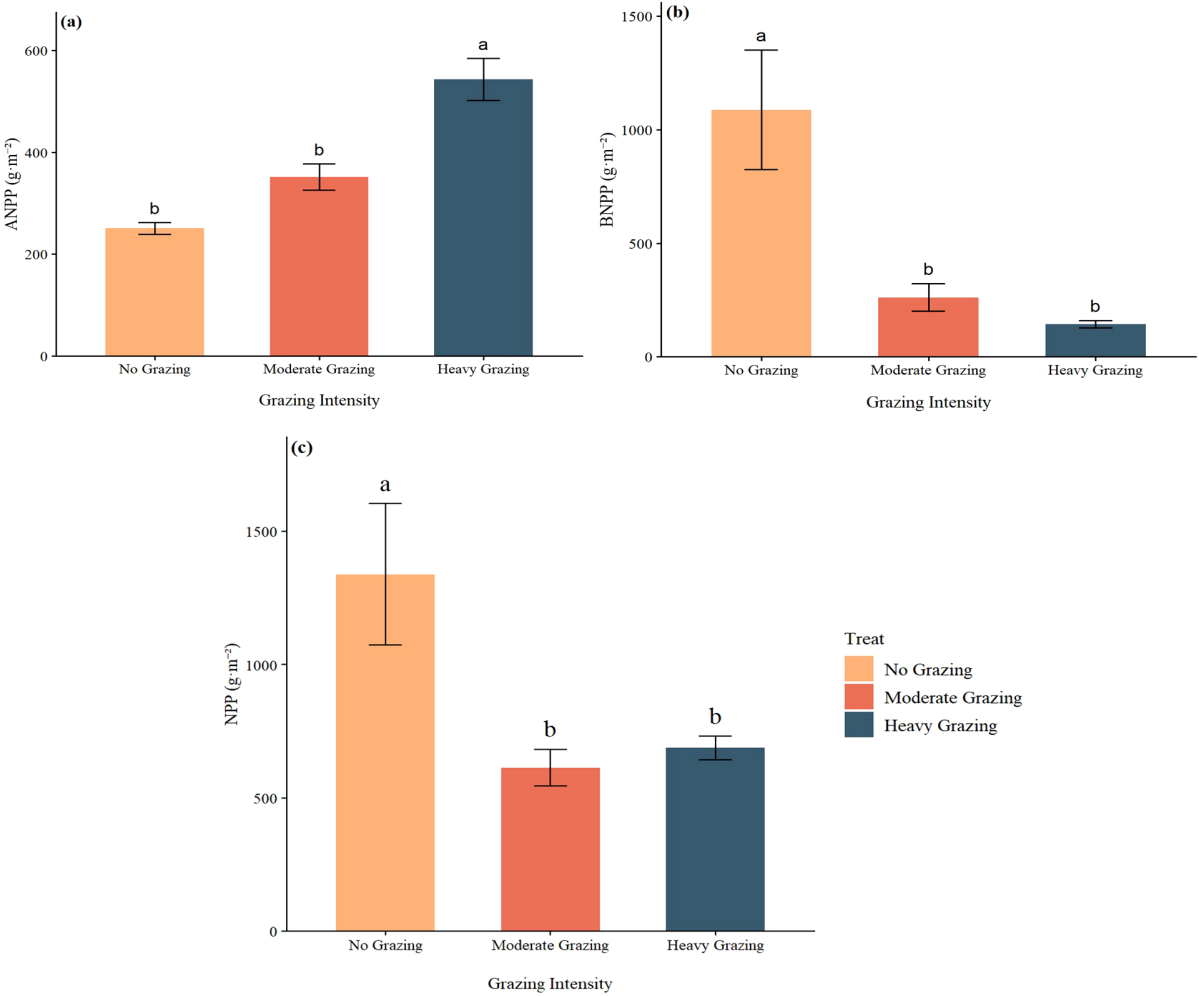
Effects of different treatments (HG, MG, NG) on aboveground net primary productivity (ANPP), belowground net primary productivity (BNPP), and total net primary productivity (NPP).

Grazing intensity exerted a highly significant effect on aboveground net primary productivity (ANPP) (F_2,42_ = 27.21, *P* < 0.001) (**Figure 4A**). The ANPP in heavily grazed (HG) plots was the highest (543 ± 41 g·m^-^²), significantly exceeding that in moderately grazed (MG, 352 ± 26 g·m^-^², *P* < 0.001) and ungrazed (NG, 249 ± 11 g·m^-^², *P* < 0.001) plots. Although ANPP in MG plots was significantly higher than in NG (*P* = 0.038), it was 35.2% lower than in HG. Tukey’s HSD test confirmed significant differences between all treatment pairs (NG vs. MG: *P* = 0.038; NG vs. HG: *P* < 0.001; MG vs. HG: *P* < 0.001) (**Figure 4**). Belowground net primary productivity (BNPP) was also significantly influenced by grazing intensity (F_2,42_ = 9.69, *P* = 0.0003) (**Figure 4B**). The highest BNPP was observed in ungrazed (NG) plots (1015 ± 255 g·m^-^²), which was significantly greater than in heavily grazed (HG: 144 ± 16.5 g·m^-^², *P* = 0.0006) and moderately grazed (MG: 262 ± 61.2 g·m^-^², *P* = 0.003) plots. However, no significant difference was detected between MG and HG plots (p = 0.848) (**Figure 4**). It is noteworthy that BNPP displayed a marked decreasing trend with increasing grazing intensity, following the order NG > MG > HG, with an 85.8% reduction from NG to HG, indicating that belowground biomass accumulation is highly sensitive to grazing disturbance. A significant effect of grazing intensity was also detected on total net primary productivity (NPP) (F_2,42_ = 5.18, *P* = 0.010) (**Figure 4C**). Ungrazed (NG) plots exhibited the highest NPP (1264 ± 258 g·m^-^²), significantly surpassing that of heavily grazed (HG: 687 ± 43.6 g·m^-^², *P* = 0.033) and moderately grazed (MG: 614 ± 68.4 g·m^-^², *P* = 0.014) plots. No significant difference was found between MG and HG plots (*P* = 0.941) (**Figure 4**). The overall pattern of NPP was NG > HG ≈ MG, with NG being 45.7% and 51.4% higher than HG and MG.

**Figure 5:**
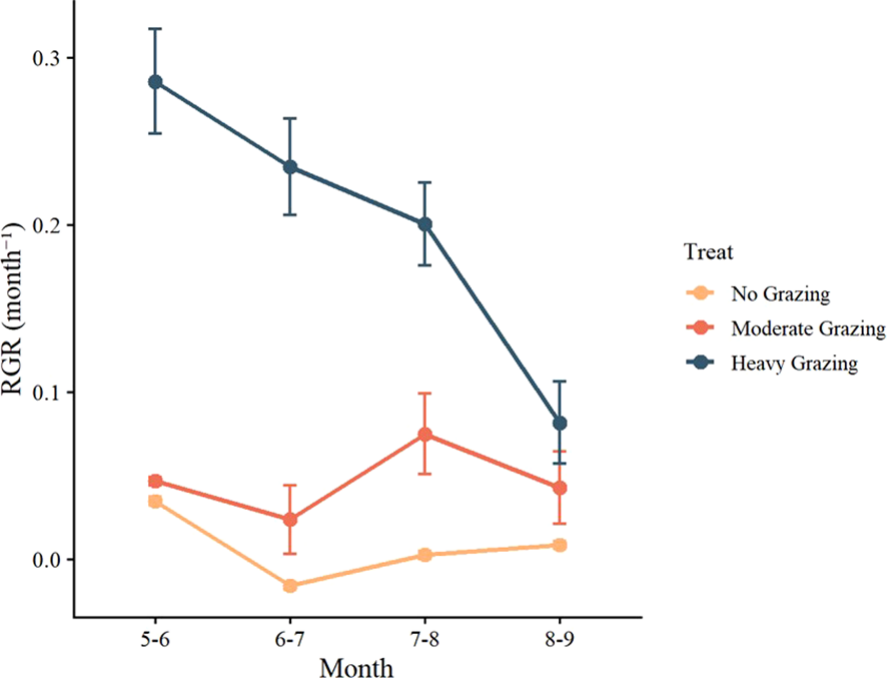
Effects of grazing intensity on the relative growth rate (RGR).

Grazing intensity and seasonal variation significantly influenced the relative growth rate (RGR) of the herbaceous marsh plant community (**Figure 5**). Repeated-measures Grazing intensity and seasonal variation had significant effects on the relative growth rate (RGR) of herbaceous marsh wetland communities (**Figure 5**). Repeated-measures ANOVA revealed a strong main effect of grazing intensity on RGR (F_2,42_ = 97.92, *P* < 0.001), with RGR under heavy grazing (HG) being significantly higher than that under moderate grazing (MG) and no grazing (NG) (*P* < 0.001). In addition, MG exhibited a significantly higher RGR than NG (*P* = 0.036). A significant main effect of season was also detected (F_3,126_ = 8.68, *P* < 0.0019), characterized by a significant difference in RGR between May–June and August–September (*P* = 0.010). Moreover, the grazing × season interaction was significant (F_6,126_ = 7.32, *P* < 0.001): in the early growing season (May–June), HG had a significantly higher RGR than both MG and NG (*P* < 0.001), whereas in the late growing season (August–September), only HG remained significantly higher than NG (*P* = 0.023).

### Dynamics of soil nutrients under different grazing intensities

**Figure 6:**
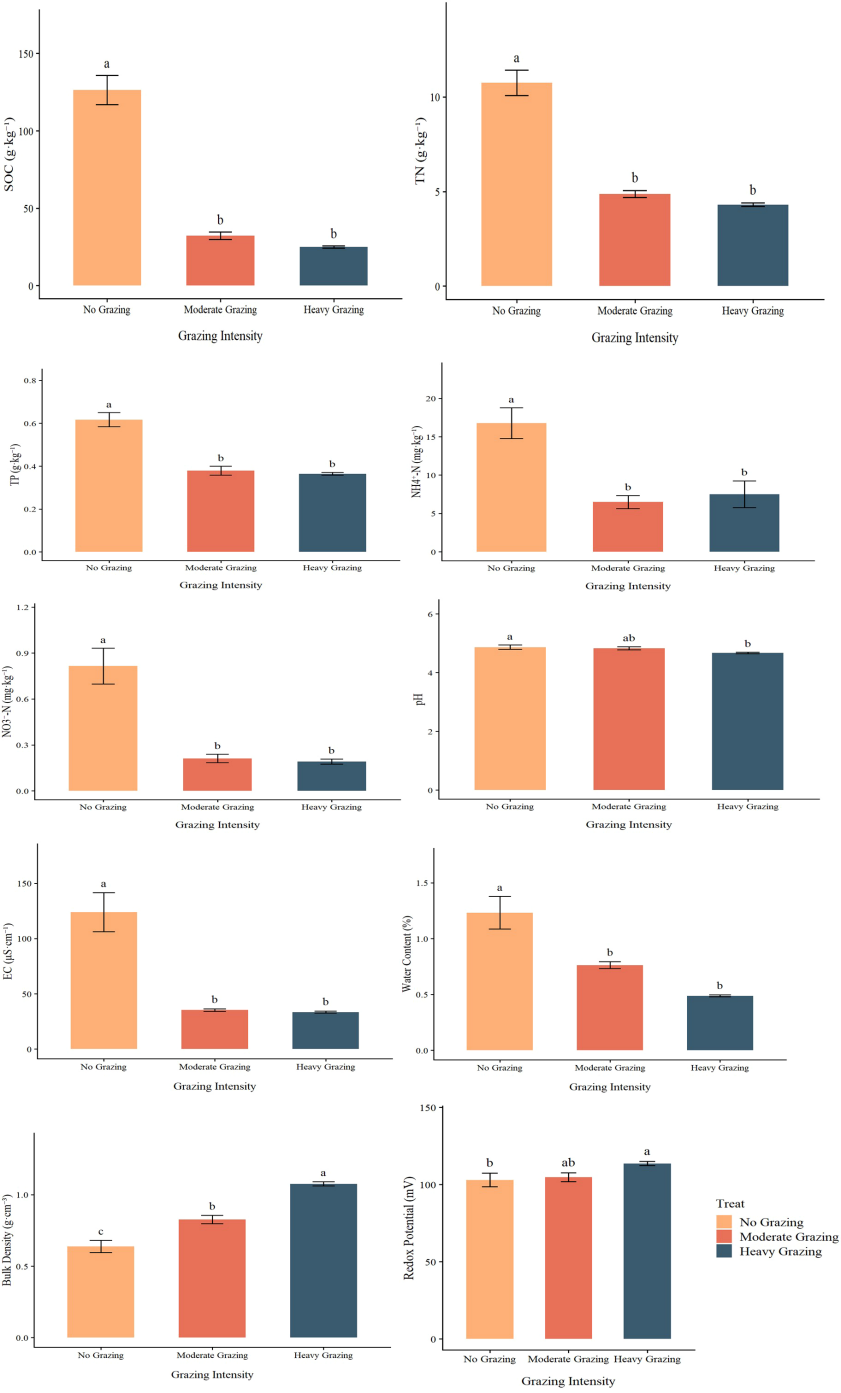
Effects of different grazing intensities on soil chemical properties. SOC, soil organic carbon; TN, total nitrogen; TP, total phosphorus; NH_4_^+^-N, ammonium nitrogen; NO_3_^-^-N, nitrate nitrogen; EC, electrical conductivity; Water content, soil water content; Bulk density, soil bulk density; Redox potential, redox potential. Different lowercase letters indicate statistically significant differences at *P* < 0.05.

Grazing intensity significantly altered the soil physicochemical properties in the herbaceous marsh wetland (**Figure 6**). Soil organic carbon (SOC), total nitrogen (TN), total phosphorus (TP), nitrate nitrogen (NO_3_^-^-N), electrical conductivity (EC), and soil water content (SWC) all exhibited a decreasing trend with increasing grazing intensity. The ammonium nitrogen (NH_4_^+^–N) content was higher in the ungrazed plots than in the grazed plots, with slightly higher values under heavy grazing than under moderate grazing. The highest concentrations of SOC (45.3 ± 2.1 g·kg^-^¹), TN (12.4 ± 0.8 g·kg^-^¹), and TP (0.65 ± 0.03 g·kg^-^¹) were observed in the no-grazing (NG) treatment. These values decreased by 57–61%, 61.5%, and 46.2% under moderate (MG) and heavy grazing (HG) intensities, respectively. NH_4_^+^-N and NO_3_^-^-N concentrations in NG were 15.3 ± 1.5 mg·kg^-^¹ and 1.05 ± 0.07 mg·kg^-^¹, respectively, which declined by over 60% and 77.8% following grazing. Reductions in EC and SWC reached 73.1–80.8% and 35.7–42.9%, respectively. In contrast, soil bulk density and oxidation-reduction potential (ORP) increased significantly with grazing intensity. Under HG, bulk density (1.05 ± 0.06 g·cm^-^³) was 75.0% and 41.7% higher than that in NG (0.62 ± 0.03 g·cm^-^³) and MG (0.78 ± 0.04 g·cm^-^³), respectively. ORP in HG (132.5 ± 6.4 mV) was 21.1% higher than in NG (118.6 ± 5.8 mV).

### Pathways through which grazing regulates above- and belowground productivity via species diversity and soil environment

**Figure 7:**
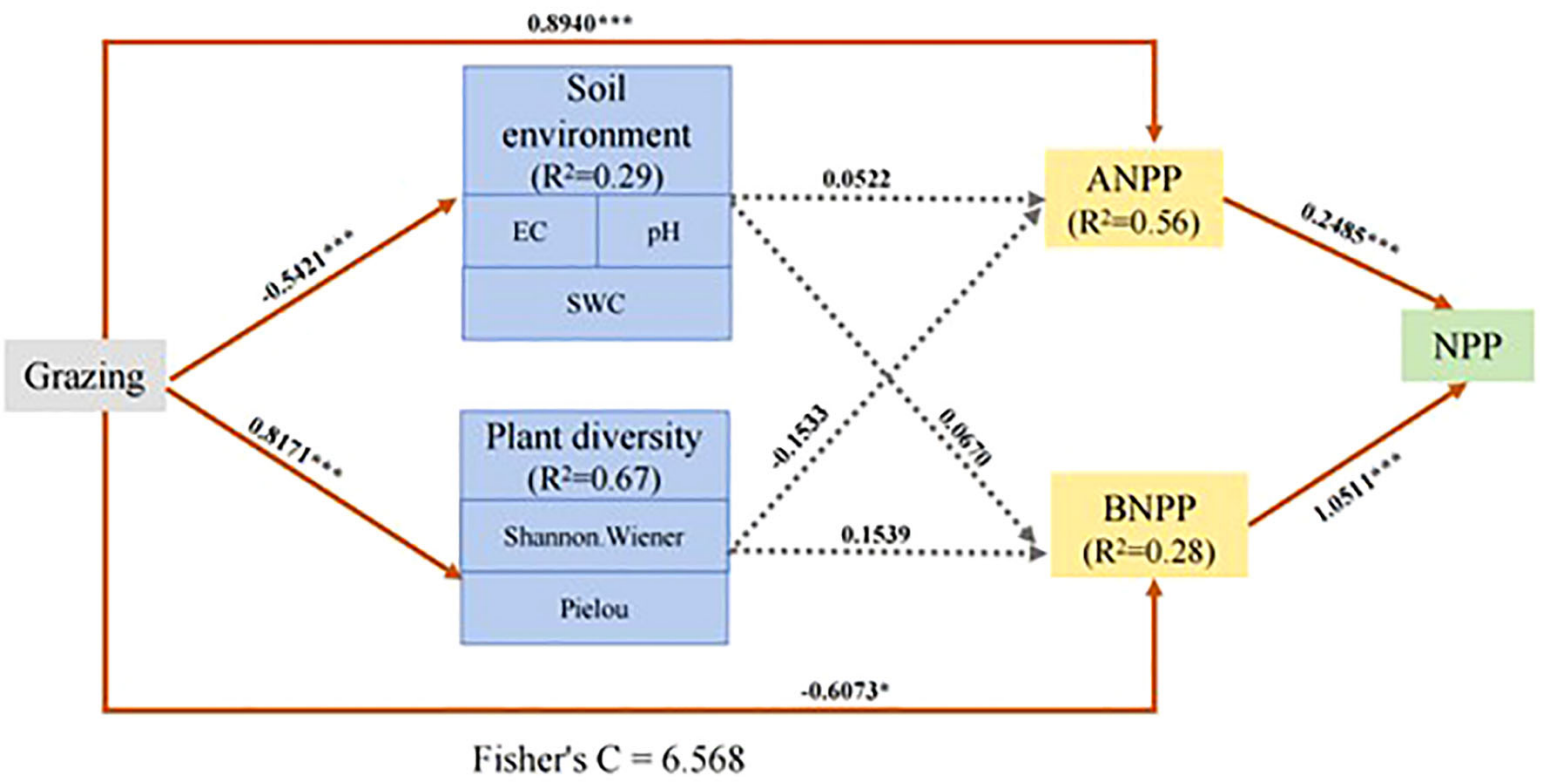
Structural equation model illustrating the direct and indirect effects of grazing on aboveground (ANPP), belowground (BNPP), and total net primary productivity (NPP) through biodiversity and the soil environment. Solid lines indicate significant paths (*P* < 0.05), while dashed lines represent non-significant paths (*P* > 0.05). Numbers adjacent to arrows denote standardized path coefficients. Model fit indices: Fisher’s C, 6.568; *P*, 0.584; AIC, –1565.041; The final model demonstrates good overall fit.

To clarify the pathways through which grazing affects wetland primary productivity, we constructed a structural equation model (SEM) incorporating grazing intensity, plant diversity indices, soil physicochemical properties, aboveground net primary productivity (ANPP), belowground net primary productivity (BNPP), and total net primary productivity (NPP) (**Figure 7**). The final model showed a good fit (Chi-square = 3.462, df = 4, *P* = 0.484; Fisher’s C = 6.568, df = 8, *P* = 0.584; AIC = –1565.041), indicating reliable explanatory capacity. Model results revealed that grazing intensity exerted a significant positive direct effect on ANPP (standardized path coefficient = 0.8940, *P* < 0.001) and a significant negative direct effect on BNPP (–0.6073, *P* < 0.05), suggesting a trade-off in above–belowground resource allocation under grazing pressure. Grazing also had a strong positive effect on plant diversity (0.8171, *P* < 0.001) but significantly reduced soil nutrient status (–0.5421, *P* < 0.001), mainly reflected in changes in soil electrical conductivity (EC), soil water content (SWC), and pH. However, indirect pathways from plant diversity and soil factors to ANPP and BNPP were not significant (e.g., diversity → ANPP: –0.1533, *P* > 0.05; soil factors → BNPP: 0.0670, *P* > 0.05). Importantly, BNPP emerged as the dominant determinant of NPP (1.0511, *P* < 0.001), with ANPP exerting a secondary positive effect (0.2485, *P* < 0.001). The model explained 67% of the variation in plant diversity, 29% in soil factors, 56% in ANPP, 28% in BNPP, and 100% in NPP, underscoring the central role of BNPP in wetland carbon cycling. Collectively, the SEM results demonstrate that grazing influences ANPP and BNPP primarily through direct pathways, thereby regulating NPP dynamics, while the mediating roles of plant diversity and soil environment were comparatively weak. .

## Discussion

### Grazing intensity drives changes in plant community structure and species diversity

Our study demonstrates that grazing significantly alters plant community diversity indices (e.g., Shannon–Wiener diversity index, Pielou evenness index, and Margalef richness index) and community composition, with grazing intensity being the primary driving factor. Under natural no-grazing conditions, a significant negative correlation was observed between plant community diversity and grazing intensity. This phenomenon can be largely attributed to the fact that in highly fertile and nutrient-rich soils, fast-growing and highly competitive plant species tend to dominate. These species rapidly absorb resources and occupy growing space, thereby suppressing the development of other species and reducing biodiversity in the absence of grazing. Such patterns have been documented in recent grassland studies showing that intense grazing disturbance alters species dominance and reduces α diversity, and that the removal of grazing pressure (e.g., grazing exclusion) can lead to competitive dominance of a few prolific species that reduce overall community diversity (Lv et al., 2024; Wang et al., 2025), which posits that competition intensifies in resource-rich environments, often resulting in plant community structures dominated by superior competitors such as species from *Cyperaceae* or *perennial Poaceae* families. These groups dominate community composition through vertical structural advantages and high photosynthetic efficiency, ultimately leading to reduced plant diversity.

Under moderate grazing conditions, our findings are consistent with recent empirical support for the intermediate disturbance hypothesis (IDH), which predicts that moderate levels of disturbance reduce competitive exclusion by dominant species, thereby promoting niche differentiation and facilitating coexistence of more species in grassland communities (Lv et al., 2024). Moderate grazing can prevent the monopolization of resources by a few dominant taxa and maintain species turnover, resulting in higher diversity under intermediate disturbance levels. In contrast, under heavy grazing intensity, excessive herbivory markedly reduces plant height, overall biomass, and the abundance of palatable species, leading to significant shifts in dominant species and lower diversity. This pattern has been observed in long-term grazing experiments showing that heavy grazing leads to loss of subordinate species and simplification of plant communities (Li et al., 2024). Furthermore, the expansion of degraded and invasive species, such as *Humulus scandens* and *Ambrosia artemisiifolia*, alters the original plant community structure, resulting in notable declines in both species richness and evenness. Additionally, under moderate grazing, *graminoids* (*Poaceae*) and *sedges* (*Cyperaceae*) are gradually replaced by *forbs* as the dominant functional group. This shift may be attributed to the greater adaptability and habitat plasticity of forbs in disturbed environments.

### Differential responses and trade-offs of aboveground, belowground, and total net primary productivity to grazing intensity

Our findings demonstrate that aboveground net primary productivity (ANPP) and belowground net primary productivity (BNPP) exhibit distinct responses under different grazing intensities, revealing a typical trade-off relationship. Specifically, ANPP increased significantly with increasing grazing intensity, whereas BNPP decreased markedly. This contrasting response of ANPP and BNPP supports the idea of resource allocation trade-offs in grassland plants, where grazing pressure alters the balance of carbon allocation between aboveground and belowground compartments (Hu et al., 2025; Zhang et al., 2025). Such allocation shifts likely reflect adaptive strategies to optimize light capture and regrowth under defoliation, at the cost of root production and belowground investment.

In our study, both moderate (MG) and heavy grazing (HG) significantly enhanced aboveground productivity, likely due to the activation of strong compensatory growth mechanisms under grazing pressure (McNaughton, 1983). First, grazing improves plant nutrient uptake and redistribution efficiency. Grazing is often accompanied by the return of animal excreta (e.g., dung and urine), which increases short‐term nitrogen availability in the topsoil, thereby enhancing plant nitrogen absorption capacity and promoting chlorophyll synthesis and photosynthetic rates (Bardgett and Wardle, 2003; Frank and McNaughton, 1993). Second, grazing reduces interspecific competition and releases resources, particularly light. By preferentially removing the aboveground biomass of dominant or tall species (e.g., perennial grasses), grazing provides more light opportunities for low‐stature or early‐season species, facilitating their rapid growth (Belsky, 1986; Holland and Detling, 1990). This process enhances community photosynthetic activity and promotes plant diversity, thereby increasing ecosystem productivity redundancy and resilience (Hector et al., 1999). Furthermore, defoliation stimulates plants to increase water and nutrient uptake rates, reshape root–shoot ratios, and enhance the functionality of resource‐acquiring tissues (Briske, 1996). This phenomenon has also been reported in ecosystems such as alpine meadows on the Qinghai–Tibet Plateau (He et al., 2025). Notably, ANPP exhibited a pattern of HG > MG > NG, which contrasts with the conventional ecological understanding that grazing suppresses productivity, this pattern may partly reflect grazing-induced compensatory growth; however, it is also possible that variation in local environmental conditions and grazing-associated microsite heterogeneity contributed to the observed differences in ANPP.

However, BNPP decreased significantly under heavy grazing, indicating suppressed root growth. This may result from grazing‐induced reallocation of resources, whereby plants prioritize limited photoassimilates to rapidly rebuild aboveground tissues to sustain survival and reproduction, at the expense of investment in belowground systems (i.e., root growth and maintenance) (Bardgett et al., 2014; Wardle et al., 2004). Moreover, although ANPP was highest under heavy grazing, total net primary productivity (NPP) was not significantly greater than that in ungrazed (NG) plots and was even slightly lower. This result highlights the critical role of BNPP in maintaining overall NPP and supports the “belowground priority hypothesis” — that belowground productivity contributes more substantially to total NPP than aboveground productivity in many natural grasslands, especially under grazing or drought stress (Steinauer and Collins, 2001; Yang et al., 2009). Overemphasizing changes in ANPP may lead to an overestimation of the positive effects of grazing on overall ecosystem productivity, while neglecting its potential inhibitory effects on belowground ecological processes. Therefore, assessing the impact of grazing on plant community net primary productivity requires integrated consideration of both aboveground and belowground components.

Temporal dynamics in relative growth rate (RGR) further corroborated this trade‐off. Heavily grazed (HG) plots exhibited significantly higher RGR in the early to mid‐growing season (May–July), indicating strong growth activity likely due to rapid recovery of leaf and stem tissues to enhance resource acquisition after grazing (Briske, 1991). However, RGR decreased markedly by August–September, reflecting that accumulated grazing pressure had exceeded the plants’ self‐repair capacity. This “early‐stimulation and late‐decline” dynamic pattern is commonly observed in grassland grazing experiments (Briske, 2017). In contrast, ungrazed (NG) plots maintained lower overall RGR, with negative values even observed in June–July, suggesting possible vegetation senescence due to competitive pressure or environmental constraints. This low but stable growth pattern likely helped maintain higher BNPP, thereby contributing to the advantage in total NPP. Thus, although aboveground growth was slower in NG plots, greater belowground accumulation resulted in higher NPP over the entire growing season.

In conclusion, grazing intensity shapes a clear trade‐off between aboveground and belowground productivity by modulating plant resource allocation strategies. Heavy grazing induces plants to allocate more carbon to aboveground tissues to compensate for losses, but at the cost of reduced investment in root systems, thereby lowering BNPP and limiting the enhancement of overall system productivity. Consequently, in formulating grassland management and ecological restoration strategies, it is essential to avoid focusing solely on short‐term increases in aboveground biomass while neglecting the sustainability of belowground carbon cycles and soil ecological processes.

### Mechanisms of grazing effects on soil nutrients and physicochemical properties

Our study indicates that grazing exerts complex influences on soil nutrient dynamics through mechanisms such as plant-soil feedbacks, litter decomposition, and animal-mediated nutrient redistribution, leading to significant alterations in soil organic matter content, with grazing intensity serving as the primary driver. The present study found that moderate grazing significantly modified soil inorganic nitrogen forms and reduced electrical conductivity (EC), suggesting that grazing to some extent activated soil nutrients and improved the ionic environment. Soil available nitrogen, including both NH_4_^+^-N and NO_3_^-^-N, was significantly higher under no grazing than under grazing treatments (both moderate and heavy), indicating that grazing decreased soil inorganic N pools. Grazing reduced plant biomass and litter input, which can limit the substrates available for microbial mineralization and nitrification processes—key pathways for generating available inorganic nitrogen (Hamilton and Frank, 2001; Shan et al., 2011).Livestock trampling is well known to increase soil bulk density and reduce soil porosity, which constrains soil aeration and microbial activity and thereby inhibits the transformation of organic nitrogen into inorganic NH_4_^+^ and NO_3_^-^ (Shan et al., 2011). These changes in soil physical properties can suppress net nitrogen mineralization rates and reduce overall nitrogen availability in grazed grasslands. Although herbivore excreta can locally input inorganic N to the soil surface, numerous studies have shown that grazing often results in net decreases in soil N pools and availability at the ecosystem scale, especially under moderate and heavy grazing, due to enhanced plant uptake, volatilization, leaching losses, and reduced microbial turnover (Bai et al., 2010; Zhu et al., 2025). The decrease in EC under grazing conditions may be caused by reduced evapotranspiration and enhanced infiltration resulting from decreased plant cover, promoting salt leaching; it may also be associated with rhizosphere processes and ion transport (Cipriotti and Aguiar, 2005; Reddy et al., 2022). No significant changes in pH were observed, possibly owing to the strong buffering capacity of the wetland soil and counterbalancing acid-base effects generated during nitrogen transformation processes (Kozlowski, 1997). In this study, soil organic carbon (SOC) responded significantly to variations in grazing intensity, showing a pronounced decline under increasing grazing pressure. This finding is consistent with previous studies demonstrating that grazing disturbance can reduce SOC through enhanced biomass removal, soil physical disturbance, and accelerated decomposition processes ( (Milchunas and Lauenroth, 1993). Overall, moderate grazing enhances soil nutrient supply capacity—particularly inorganic nitrogen—without causing significant detriment to SOC or pH, supporting the concept of “grazing-induced nutrient activation” (Hobbs, 1996). However, heavy grazing disrupts this balance, indicating the existence of an intensity threshold for nutrient maintenance. Therefore, in wetland grazing management, intensity should be properly controlled to achieve a balance between ecosystem productivity and nutrient stability.

### Regulatory effects of grazing intensity, plant species diversity, and soil properties on net primary productivity via different mechanisms

Our study revealed that grazing intensity, plant species diversity, and soil nutrients significantly influenced net primary productivity (NPP). Grazing directly drove both aboveground net primary productivity (ANPP) and belowground net primary productivity (BNPP), thereby affecting total NPP. In contrast, plant species diversity and soil nutrients, acting as mediating variables, exhibited non-significant path effects. Although plant diversity and soil environment are often regarded as important drivers of ecosystem functioning (Tilman et al., 1997), their actual regulatory roles appear constrained by interactions among factors such as disturbance intensity, temporal scale, and niche stability. Regarding the relationship between species diversity and primary productivity, conventional theories posit that biodiversity enhances ecosystem resource-use efficiency and stability through mechanisms such as complementarity effects and redundancy effects (Hooper et al., 2005; Loreau and Hector, 2001). However, our results showed that the indirect path coefficients of plant species diversity on both ANPP and BNPP were non-significant, indicating that diversity did not contribute significantly to productivity through mechanisms such as resource complementarity or dominant species replacement. A possible explanation lies in the specialized plant assemblages and long-term grazing-disturbed community structures in wetland ecosystems, where high niche overlap may limit the realization of functional complementarity among species (Laliberte et al., 2014). Furthermore, although invasive or highly adaptable forb species may increase species richness and diversity indices (e.g., Shannon–Wiener index), their ecological functions are often singular and do not substantially enhance carbon sequestration capacity (Flombaum and Sala, 2008).Soil organic carbon (SOC), available nitrogen (NH_4_^+^-N, NO_3_^-^-N), pH, and electrical conductivity (EC) are key factors influencing plant nutrient uptake and growth (Van Der Putten et al., 2013). To reconcile the discrepancy between the nonsignificant direct soil–productivity pathways identified by the structural equation model and the significant treatment effects on soil physicochemical properties revealed by ANOVA, we further explored the underlying mechanisms. These contrasting results likely arise from the different conceptual roles of the two analytical frameworks: ANOVA characterizes differences in soil properties among grazing treatments, whereas SEM evaluates the relative contribution of soil variables to productivity through direct causal pathways. In this context, significant variation in soil properties does not necessarily translate into strong direct control over productivity. One possible explanation is that soil conditions, although altered by grazing, did not act as the primary limiting factors for productivity, with climatic conditions or grazing management exerting stronger regulatory effects (Bardgett et al., 2021). Alternatively, soil influences on productivity may be mediated through indirect pathways, including shifts in microbial community structure, nutrient transformation processes, or changes in root physiological traits (Bardgett and Caruso, 2020). Elucidating these indirect pathways will be essential for a more mechanistic understanding of soil–productivity linkages under grazing disturbance. Nevertheless, our analysis indicated that the path coefficients of soil factors on both BNPP and ANPP were low and non-significant. This suggests that short-term grazing-induced changes in soil physicochemical properties are not sufficient to significantly alter plant productivity patterns. On one hand, marsh wetlands possess strong hydrological buffering and nutrient retention capacities, leading to lagged responses to external disturbances (Reddy et al., 2023). On the other hand, although grazing-induced increases in bulk density and redox potential alter the root zone environment, associated constraints on root growth may be mitigated through plant physiological mechanisms such as root–shoot ratio adjustments (Bardgett and Wardle, 2003). Notably, the structural equation model (SEM) results showed that the standardized path coefficient from BNPP to total NPP was much higher than that from ANPP, indicating that the belowground compartment is the key determinant of overall carbon accumulation and ecosystem functioning in wetland environments. This finding supports the belowground priority hypothesis, which suggests that in herbaceous ecosystems—particularly under disturbance or limiting conditions—productivity is largely sustained by the root system (De Deyn et al., 2008; Jackson et al., 1997). This also explains why, under heavy grazing, although ANPP increased significantly, NPP did not exhibit an overall increase due to a pronounced decline in BNPP. Additionally, the SEM explained 67% of the variance in species diversity, 29% in soil properties, 56% in ANPP, 28% in BNPP, and 100% in NPP. Although the model showed good fit (AIC = –1565.041), the explanatory power of soil and diversity variables remained relatively limited. This further emphasizes that in herbaceous marsh ecosystems characterized by high disturbance levels and specific hydrological conditions, traditional “diversity–function” hypotheses may not be fully applicable. Instead, mechanistic understanding requires consideration of system-specific contexts (Fridley, 2003; Grace et al., 2007). Therefore, future research should place greater emphasis on: 1) the coupling between changes in community functional traits and productivity; 2) the dynamic responses of soil nutrients and microbial communities over longer time scales; and 3) the interactive effects of grazing and hydrological variation, in order to more comprehensively assess the carbon cycle and ecological restoration potential of herbaceous wetlands under grazing disturbance.

## Conclusion

Whether grazing affects net primary productivity (NPP) of plant communities depends on the integrated response of both aboveground and belowground productivity. Grazing intensity significantly influences the primary productivity of herbaceous marsh ecosystems by regulating plant carbon allocation aboveground and belowground. Moderate grazing enhanced aboveground net primary productivity (ANPP) by triggering compensatory growth mechanisms, while heavy grazing significantly reduced belowground net primary productivity (BNPP), demonstrating a typical resource allocation trade-off. Structural equation modeling (SEM) further indicated that belowground net primary productivity (BNPP) exerted a relatively stronger influence on total NPP than aboveground productivity in the studied marsh wetland ecosystem. Although grazing significantly affected plant diversity and soil nutrient conditions, its indirect pathways influencing NPP were weak, indicating that short-term productivity changes in wetland systems are primarily driven directly by plant resource allocation strategies, rather than being mediated by species complementarity or soil factors. This study highlights the central role of belowground productivity in maintaining wetland ecosystem functioning and suggests that heavy grazing may lead to restricted root development, deterioration of soil structure, and decline in carbon sink function, thereby reducing the long-term stability of the ecosystem.

## Data Availability

The original contributions presented in the study are included in the article/supplementary material. Further inquiries can be directed to the corresponding author.
